# The potential role of hypothalamic POMC^TRPM2^ in interscapular BAT thermogenesis

**DOI:** 10.1038/s12276-025-01538-6

**Published:** 2025-09-12

**Authors:** Ju Hwan Yang, Arbi Bahtiar Boedi Iman Halanobis, Eun-Hye Byeon, Na Hyun Park, Sang Won Park, Hyun Joon Kim, Dawon Kang, Deok-Ryong Kim, Jinsung Yang, Eun Sang Choe, Wanil Kim, Dong Kun Lee

**Affiliations:** 1 Department of Physiology, Institute of Medical Sciences, Gyeongsang National University College of Medicine, Jinju, Republic of Korea; 2 Convergence of Medical Sciences, Gyeongsang National University College of Medicine, Jinju, Republic of Korea; 3 Department of Pharmacology, Institute of Medical Sciences, Gyeongsang National University College of Medicine, Jinju, Republic of Korea; 4 Department of Anatomy, Institute of Medical Sciences, Gyeongsang National University College of Medicine, Jinju, Republic of Korea; 5 Department of Biochemistry, Institute of Medical Sciences, Gyeongsang National University College of Medicine, Jinju, Republic of Korea; 6 Department of Biological Sciences, Pusan National University, Busan, Republic of Korea

**Keywords:** Hypothalamus, Ion channels in the nervous system, Obesity

## Abstract

The major function of primary order neurons in the arcuate nucleus of the hypothalamus is control of energy homeostasis. Among these neurons, proopiomelanocortin (POMC) neurons play a significant role in controlling anorexigenic feeding behavior and upregulating energy expenditure. In addition, transient receptor potential melastatin 2 (TRPM2) is a well-established temperature sensor, but no evidence of regulation of brown adipose tissue (BAT) thermogenesis via POMC^TRPM2^ neurons in the arcuate nucleus has been reported so far. Here, through single-cell reverse-transcription and immunohistochemistry analyses, we confirmed that a subset of POMC neurons express TRPM2. Also, we confirmed the neuronal connection between POMC and BAT using cholera toxin subunit B. The chemogenetic stimulation of POMC neurons induced BAT thermogenesis, and this thermogenic effect was inhibited by a TRPM2 blocker. These results indicate that TRPM2 could modulate POMC neuronal activity and play a role in regulating BAT activity through neuronal connections. Adenosine diphosphoribose (ADPR), a TRPM2 agonist, depolarized POMC neurons, and this effect was suppressed by TRP and TRPM2 antagonists. In addition, intracerebrovascular injection of ADPR increased c-Fos expression of a subset of POMC neurons, BAT and core body temperature and expression of IRF-4, but not uncoupling protein 1, in normal chow diet- and high-fat diet-fed mice. TRPM2 antagonists blocked this increase. Our findings offer new insights into the physiological mechanism of IRF-4-mediated BAT thermogenesis, which is regulated by acute activation of hypothalamic POMC^TRPM2^ neurons. Consequently, these approaches to promoting BAT thermogenesis can provide novel basic concepts to establish therapeutic strategies and precautions to combat metabolic disorders.

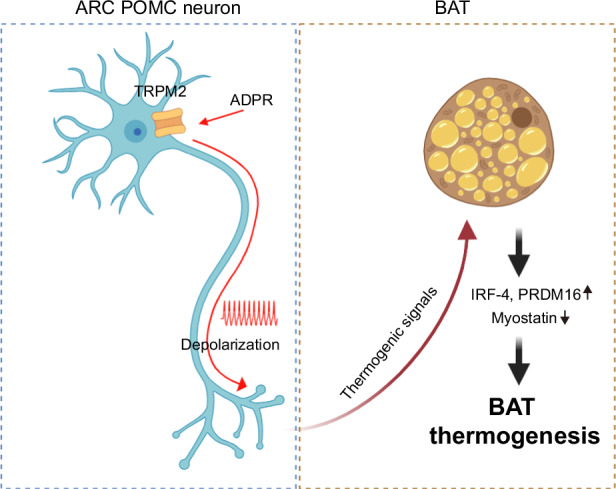

## TRPM2 neurons link hypothalamus to brown fat thermogenesis

The hypothalamus, a part of the brain, helps to control hunger and energy use by processing signals from hormones and nutrients. This study explores how certain brain cells called POMC neurons influence energy balance and body temperature. Researchers found that a protein channel called TRPM2, present in POMC neurons, plays a role in regulating body heat through brown fat. In experiments with mice, scientists used various techniques to study these neurons and their connection to brown fat. They used genetic tools to activate POMC neurons and observed changes in body temperature. The study showed that activating TRPM2 channels in these neurons increased heat production in brown fat, even in mice on a high-fat diet. The results suggest that TRPM2 channels help POMC neurons to regulate body temperature and energy use.

This summary was initially drafted using artificial intelligence, then revised and fact-checked by the author.

## Introduction

The hypothalamus regulates feeding and energy balance by integrating various inputs, including hormones, nutrients and neural signals from other brain areas^1,2^. It is pivotal in maintaining energy homeostasis through precise control of both energy intake and expenditure^3–6^. Previous studies have highlighted that, among hypothalamic neurons, anorexigenic proopiomelanocortin (POMC) and orexigenic neuropeptide Y (NPY)/Agouti-related peptide (AgRP) neurons in the arcuate nucleus (ARC) interact with each other and play essential roles in regulating energy balance, which involves both glucose homeostasis and energy consumption^7–9^. Specifically, POMC neurons promote satiety signals and restrain feeding behavior, while enhancing energy expenditure through their involvement in adipose tissue metabolism. Notably, deficiencies in the *Pomc* gene in the brain or malfunction of melanocortin receptors in POMC neurons lead to diet-induced obesity and associated metabolic disorders in mice^10–13^. Studies in transgenic mice exhibiting enhanced leptin and insulin receptor signaling in POMC neurons demonstrated improved glucose handling and increased energy expenditure^14^. By contrast, *Pomc*-knockout mice failed to increase energy expenditure or enhance physical activity after high-fat diet (HFD) feeding^15^.

Transient receptor potential (TRP) melastatin 2 (TRPM2) is a subfamily of TRP ion channels that are nonselectively permeable to cations, including calcium^16,17^. *TRPM2* mRNA is expressed across all tissues except bone and cartilage and is particularly abundant in the brain^16^. While TRPM2 is involved in various physiological processes, it also plays a crucial role in temperature sensing and thermoregulation within the hypothalamus^17–19^. Indeed, activation of TRPM2 is triggered by stimuli such as warmth, redox signals and endogenous ligands, including adenosine diphosphoribose (ADPR) and cyclic ADPR^20,21^. In particular, a subset of TRPM2-expressing neurons in the preoptic area (POA) of the hypothalamus downregulates body temperature in response to hyperthermia, contributing to temperature homeostasis^17–19^. Despite the widespread distribution of TRPM2 in the brain and its potential role in hypothalamic thermoregulation, the current understanding of its role is primarily confined to the POA.

The melanocortin system, encompassing POMC and NPY/AgRP neurons in the ARC, is crucial for maintaining energy balance^6,22^. Among the neurons of this system, POMC neurons release α-melanocyte-stimulating hormone (α-MSH), a fragment cleaved from the precursor POMC peptide, which stimulates the paraventricular nucleus (PVN) and, consequently, induces brown adipose tissue (BAT) thermogenesis^22–24^. Notably, POMC neurons mediate autonomic nerve activity, contributing to leptin-induced BAT sympathetic nerve activation^25^. Previous studies have shown that the activation of POMC by mesencephalic astrocyte-derived neurotrophic factor induces BAT thermogenesis^26^. Furthermore, POMC neurons can increase uncoupling protein-1 (UCP1) expression in the BAT by inhibiting tyrosine phosphatase-1B and T cell protein tyrosine phosphatase signaling after leptin treatment^27^. Thus, activation of POMC neurons increases energy expenditure via BAT thermogenesis. However, the role of TRPM2 expression in POMC neurons, and the precise mechanism by which it enhances BAT thermogenesis, remain unclear.

We investigated the hypothesis that TRPM2 channels expressed in POMC neurons in the ARC may be involved in BAT thermogenesis. Here, to establish the thermoregulatory role of hypothalamic POMC^TRPM2^ neurons in BAT thermogenesis, we demonstrated TRPM2 expression in ARC POMC neurons, revealed BAT–ARC neuronal connectivity and showed the regulation of BAT and core body temperature via POMC^TRPM2^ neurons in both normal chow diet (NCD)- and HFD-fed animal models.

## Materials and methods

### Animals

All experiments and animal care protocols conducted in this study received approval from the Gyeongsang National University Institution Animal Care and Use Committee (GNU IACUC, GNU-200820-M0053). The procedures were carried out in accordance with the National Institutes of Health (NIH) guidelines and adhered to a scientifically reviewed protocol (GLA-100917-M0093). The mice utilized in these experiments included POMC-Cre (stock no. 005965, Jackson Laboratory) and POMC-eGFP (stock no. 009593, Jackson Laboratory), which are mixed C57BL/6, FVB and 129 strain backgrounds. Mice were provided with either an NCD or an HFD, and water was supplied ad libitum.

### Slice preparation and electrophysiological recordings

Transverse brain slices, each with a thickness of 200 μm, were prepared using a vibratome (7000smz-2; Campden Instruments). The pipette solution contained 130 mM of K-gluconate, 5 mM of CaCl_2_, 10 mM of EGTA, 10 mM of HEPES, 2 mM of MgATP, 0.5 mM of Na_2_GTP and 10 mM of phosphocreatine. To record membrane potentials from a brain slice, the slice was placed in a recording chamber and continuously perfused with artificial cerebrospinal fluid (aCSF; containing 113 mM of NaCl, 3 mM of KCl, 1 mM of NaH_2_PO_4_, 26 mM of NaHCO_3_, 2.5 mM of CaCl_2_, 1 mM of MgCl_2_ and 5 mM of glucose in 95% O_2_/5% CO_2_) at 1.5–2 ml/min. When using brain slices from the HFD-fed mice, the glucose concentration in the aCSF was changed from 5 mM to 10 mM. The recording chambers were placed on the stage of an upright and infrared differential interference contrast microscope (Olympus BX51WI; Olympus), mounted on a Gibraltar *X*–*Y* table. The prepared brain slices were visualized using infrared microscopy with a 40× water immersion objective. Whole-cell current-clamp recordings were conducted on visually identified ARC POMC neurons of the POMC–eGFP mice brain slices at a holding potential of −70 mV. Membrane potentials were recorded in the whole-cell configuration using a MultiClamp 700B amplifier (Molecular Devices). Electrophysiological signals were low-pass filtered at 2–5 kHz, saved on a desktop personal computer and subsequently analyzed offline using pClamp 11 software (Molecular Devices). In each recording, membrane potentials measured every 30 s were considered as single data points. We compared ten data points before and after the application of drugs using paired *t*-tests. All recordings were conducted at 30 ± 2 °C.

### Immunofluorescence staining

Male mice, aged 5–6 weeks, were anesthetized using avertin and subsequently transcardially perfused with a preperfusion solution containing 10 U/ml of heparin in phosphate-buffered saline (PBS). The brains isolated from mice were incubated overnight at 4 °C in 4% paraformaldehyde dissolved in PBS. The following day, brains were serially cut into 40-μm sections using a vibratome (Leica Microsystems) and stored in 1× PBS with 0.001% sodium azide at 4 °C. For immunofluorescence staining, the slices were washed three times in 0.5% Triton-X–PBS for 10 min each. The slices were blocked with 1 ml of 0.5% Triton-X–bovine serum albumin (BSA) for 1 h, then washed three times with PBS for 10 min. The brain slices were incubated with respective primary antibodies, anti-rabbit TRPM2, anti-mouse beta subunit cholera toxin, anti-rabbit c-Fos (Abcam) or anti-mouse mCherry antibody (1:500, Novus) at 1:500 overnight with 2% BSA–PBS at 4 °C on a rolling shaker. After three washes with PBS, Alexa Fluor 594 anti-rabbit secondary antibody (1:1,000, Abcam) with 2% BSA–PBS was labeled for 2 h at room temperature. All images were acquired using an Olympus fluorescence microscope (Olympus).

### Stereotaxic surgery and drug infusion

Mice were anesthetized with avertin and placed in a stereotaxic instrument. Sterile custom guide cannulas (RWD) were implanted into the lateral ventricle at stereotaxic coordinates AP (anterior–posterior, +0.85 mm), ML (medial–lateral, +0.5 mm), and DV (dorsal–ventral, −3.0 mm) relative to bregma for intracerebroventricular (i.c.v.) injection of ADPR (1 μl of 100 μM, Sigma-Aldrich) and clotrimazole (CTM, 1 μl of 100 μM, Sigma-Aldrich) under aseptic conditions. Animals were allowed at least a week to recover from surgery.

### Measurement of BAT and core body temperature

Under isoflurane anesthesia, core body temperature and interscapular BAT temperature were measured at room temperature (24 °C). For direct measurement of BAT and core body temperatures, a flexible implantable microprobe (IT-18 or IT-21; Physitemp Instruments) was positioned beneath the BAT, while a rectal thermoprobe (RET-4; Physitemp Instruments) was inserted into the rectum. Temperature readings from two to three mice were acquired using the THERMES-USB Temperature Data Acquisition System (Physitemp Instruments). To maintain stable body temperature during anesthesia, warming pads and heat lamps were used.

### Gq-DREADD viral injection

AAV8-hSyn-DIO-hM3D(Gq)-mCherry virus (Gq-DREADD, Addgene) was injected into the ARC of POMC-Cre::POMC-GFP mice under anesthesia with avertin through stereotaxical surgery. The AAV vectors, which are expressed cre-dependently, were injected bilaterally (0.5 μl of 4 × 10^12^ vg/ml per side) using a microsyringe (Hamilton) according to the mouse brain atlas (AP, −1.3 mm; ML, ± 0.1 mm; DV, −6.0 mm). To activate Gq-DREADD-expressing POMC neurons, clozapine-*N*-oxide (CNO) was used in both patch-clamp recording and core body temperature measurement.

### Western immunoblotting

Isolated BAT tissues were incubated in RIPA buffer with protease inhibitor cocktail (Thermo Scientific, Rockford, IL, USA) and homogenized using Beadbug microtube homogenizer (Sigma-Aldrich) at the highest frequency until clear. During homogenization, samples were kept on the ice. The homogenized samples were centrifuged at 6,000*g* for 15 min at 4 °C. After centrifugation, the pipette tip was passed through the fat cake to collect only the lysates carefully and transferred to a new tube. Triton-X was added to the final concentration of 10% and then incubated for 1 h at 4 °C. Finally, samples were centrifuged twice at 12,000*g* for 15 min at 4 °C, and the supernatants were collected. The concentrations of solubilized proteins in the supernatants were determined using a Bradford protein assay (Bio-Rad). Proteins in supernatants (10 μg) were separated using 10% sodium dodecyl sulfate–polyacrylamide gel electrophoresis. The separated proteins were transferred to a methanol-activated polyvinylidene difluoride membrane (Merck). The membrane was blocked with a blocking buffer containing 5% skim milk in a mixture of Tris-buffered saline and 0.1% Tween-20 and washed three times for 10 min. Membranes were then labeled with either a rabbit or mouse primary antiserum against UCP1 (1:20,000, Abcam), transmembrane monocarboxylate transporter 1 (MCT1, 1:1,000, Thermo Scientific), PRDM16 (1:1,000, Abcam), peroxisome proliferator-activated receptor-gamma coactivator 1-alpha (PGC1-1α, 1:1,000, Abcam), growth differentiation factor-8 (GDF8, 1:1,000, Abcam) or interferon regulatory factor 4 (IRF-4, 1:1,000, Santa Cruz Biotechnology), overnight at 4 °C, rewashed three times and incubated with horseradish peroxidase-labeled goat anti-rabbit or mouse secondary antiserum (1:1,000) (Thermo Fisher Scientific) for 1 h at room temperature. Immunoreactive protein bands were detected using an iBright western blot imaging system (Thermo Scientific) with enhanced chemiluminescence reagents, Westsave Up (1:500 ratio of reagents A to B; Ab Frontier). The same membrane was stripped and probed with mouse primary antiserum against β-actin (1:5,000) (Sigma-Aldrich) to normalize the blots. Immunoreactive protein bands were semi-quantified using a digital imaging camera and NIH Image 1.62 software.

### Single-cell RT–PCR

Single POMC neuron samples were obtained through aspiration into the patch pipette from brain slices prepared using the same method used for patch-clamp recording. The reverse transcription (RT) reaction to obtain cDNA was performed using the REPLI-g WTA single-cell kit (Qiagen). The samples, comprising total RNA from single POMC neurons in the glass pipette, were expelled into a microcentrifuge tube containing lysis buffer (with a total volume of 5.5 μl, consisting of 2 μl lysis buffer and 2.5 μl single-cell sample). The mixture was then incubated at 24 °C for 5 min and cooled to 4 °C. Subsequently, the samples were incubated for 10 min at 42 °C with 1 μl gDNA wipeout buffer before the addition of 3.5 μl RT mix (comprising 0.5 μl oligo (dT) primer, 2 μl RT buffer, 0.5 μl random primer and 0.5 μl RT enzyme mix). The tubes were incubated at 42 °C for 1 h, followed by a 3 min incubation at 95 °C. Next, the tubes were incubated at 24 °C for 30 min with 5 μl ligation mix (composed of 4 μl ligase buffer and 1 μl ligase mix). The reaction was terminated by incubating at 95 °C for 5 min. After adding the amplification mix (14.5 μl buffer and 0.5 μl DNA polymerase), samples were incubated at 30 °C for 2 h and at 65 °C for 5 min. The RT–PCR amplicon obtained from a single POMC neuron was used for PCR analysis, confirming the expression of TRPM2 and TRPM3 mRNAs through 2% agarose gel electrophoresis.

### Statistics

Statistical analyses for western blotting, real-time PCR and immunofluorescence were performed using a one-way analysis of variance with Tukey’s multiple-comparison test. Patch-clamp recording and temperature data were analyzed with paired *t*-tests. All statistical analyses were performed with GraphPad Prism 9.5.1 software (GraphPad Software). Data were considered significantly different when the *P* value was <0.05. All statistical results are presented as mean ± s.e.m.

## Results

### A subset of POMC neurons in the ARC express TRPM2 channels

**Fig. 1:**
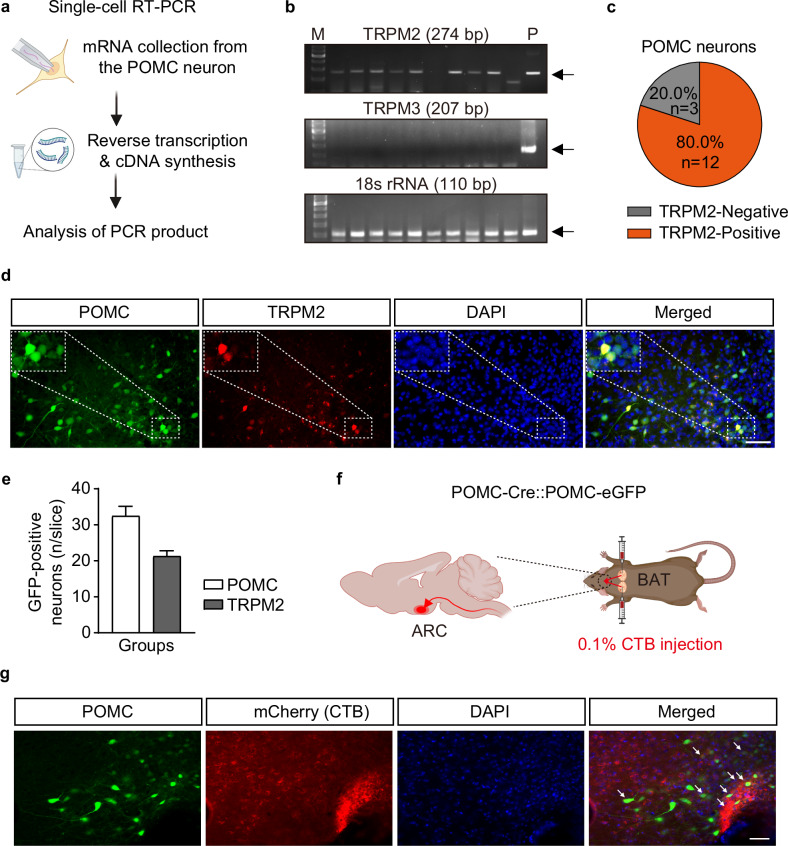
ARC POMC neurons express TRPM2 channels and innervate the interscapular BAT. **a** A schematic illustration of single-cell RT–PCR. **b** Representative images showing the expression of *Trpm2*, *Trpm3* and *18s* rRNA mRNAs in POMC neurons. **c** Percentile analysis of the TRPM2 and TRPM3 expression in ARC POMC neurons. **d** Fluorescence microscopy images showing TRPM2 channel (red) expression in the ARC of POMC-cre::POMC-eGFP mice (white arrows). Scale bar, 50 μm. **e** The average number of total POMC neurons (white bar) and TRPM2-positive POMC neurons (gray bar) in the hypothalamus from four mice (*n* = 14 slices). **f** A schematic diagram illustrating retrograde neuronal tracing strategy using CTB injection into the BAT. CTB was directly injected into both sides of the BAT of POMC-cre::POMC-eGFP mice. **g** Fluorescence microscopy images showing colocalization of a subset of POMC neurons and CTB (white arrows). Scale bar, 50 μm.

Among the TRPM subfamily, TRPM2 and TRPM3 have been demonstrated to be thermosensation channels in the brain^28–30^. In addition, TRPM2 and TRPM3 are involved in alterations of neuronal membrane potential (depolarization)^31^. To determine whether TRPM2 is expressed in POMC neurons, we conducted single-cell RT–PCR. We collected the total mRNA in the cytoplasm of individual POMC neurons using glass pipettes for patch-clamp recording and then analyzed the *Trpm2* and *Trpm3* gene expression profiles (Fig. 1a, b). As shown in Fig. 1a–c, among the POMC neurons used in the experiment, 80% were *Trpm2* positive, but all POMC neurons were *Trpm3* negative (Fig. 1b, c).

Immunofluorescence microscopy images showed that 65.5% of TRPM2 proteins were also expressed in the ARC POMC neuron subset (Fig. 1d, e). These data suggest that a subset of POMC neurons may be readily regulated by TRPM2, but not TRPM3, activity.

### The neuronal connection between ARC POMC neurons and BAT

Several studies have proposed that activation of the PVN by ARC POMC neurons within the melanocortin system plays a crucial role in BAT thermogenesis via the autonomic nervous system^6,22^. Before investigating whether POMC^TRPM2^ regulates BAT thermogenesis, we examined the neural connection between ARC POMC neurons and BAT by using the monosynaptic retrograde neuronal tracer cholera toxin subunit B (CTB, 0.1%). Two weeks after bilateral administration of CTB into the BAT of POMC-cre::POMC-eGFP mice, a subset of POMC neurons was labeled with an anti-CTB antibody (Fig. 1f, g). By this neuronal tracing study, we demonstrated the existence of CTB-positive ARC POMC neurons, which provides direct evidence for synaptic innervation of BAT by ARC POMC neurons.

### Chemogenetic modulation of POMC neuronal activity and consequent BAT thermogenesis

**Fig. 2:**
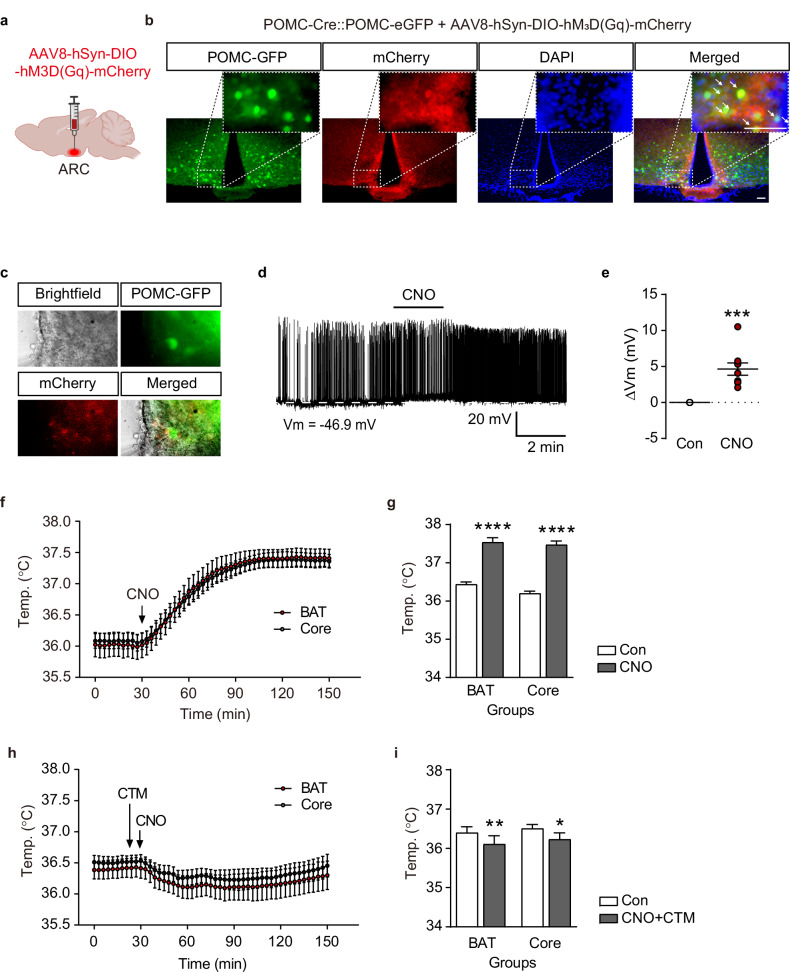
ARC POMC neuron-specific activation increases BAT activity. **a** A schematic illustration of Gq-DREADD injection into the ARC. **b** Representative images show the expression of Gq-DREADD in ARC POMC neurons of POMC-cre::POMC-eGFP mice (white arrows). Scale bar, 50 μm. **c** Representative brightfield, infrared and merged images for establishing a whole-cell patch-clamp recording after Gq-DREADD infection of ARC POMC neurons. **d** Representative recording trace for changes in membrane potential of POMC neurons after intraperitoneal. injection of CNO (10 μM). Scale bar, 20 mV, 2 min. **e** Pooled data showing POMC neuron depolarization after CNO treatment. **f** Pooled data showing alterations in both BAT and core body temperatures after i.p. injection of CNO. **g** The plot shows changes in the mean value of both the BAT and core body temperature at 0 min (white bar) and 90 min (gray bar) after CNO treatment, respectively. **h** Pooled data showing alterations in both BAT and core body temperatures after i.p. injection of CNO in the presence of CTM. **i** Plot showing changes in the mean value of both the BAT and core body temperature at 0 min (white bar) and 90 min (gray bar) after CNO treatment in the presence of CTM, respectively. All data are shown as mean ± s.e.m. *P < 0.05, **P < 0.01, ***P < 0.001, ****P < 0.0001 versus control.

Before determining whether POMC^TRPM2^ regulates BAT thermogenesis, a neural connection between ARC POMC neurons and BAT was visualized using CTB (Fig. 1e, f). Then, to determine whether ARC POMC neurons are involved in BAT thermogenesis, Gq-DREADDs were expressed in POMC neurons by bilateral injection of the AAV8-hSyn-DIO-hM3D(Gq)-mCherry virus into the ARC of POMC-cre::POMC-eGFP mice (Fig. 2a, b). All DREADD-expressing POMC neurons used in the experiment were depolarized (nine of nine neurons) after a DREADD agonist, CNO (10 μM) treatment (Fig. 2c–e). These whole-cell patch-clamp results suggest that Gq-DREADD infection is sufficient to modulate POMC neuronal activity. Then, to investigate BAT thermogenesis by activating ARC POMC neurons, CNO was i.p. injected (2 mg/kg) into Gq-DREADD-infected mice. As shown in Fig. 2f, g, both BAT (from 36.4 ± 0.2 °C to 37.5 ± 0.3 °C) and core body temperature (from 36.2 ± 0.2 °C to 37.5 ± 0.3 °C) were increased after CNO injection (*n* = 8). In addition, this increase in BAT (from 35.8 ± 0.1 °C to 35.2 ± 0.2 °C) and core body temperature (from 36.1 ± 0.1 °C to 35.5 ± 0.2 °C) by chemogenetic POMC neuronal activation was entirely diminished by pretreatment of the TRPM2 blocker CTM (50 μM, *n* = 9). As shown in Supplementary Fig. 6, silencing TRPM2 function by injecting TRPM2 siRNA into the ARC blocked POMC-induced BAT thermogenesis mediated by Gq-DREADDs (*n* = 5). These results suggest that POMC neuron-targeted stimulation could sufficiently generate heat in the BAT, and it strongly suggests that this effect is regulated by TRPM2 channels of the hypothalamic POMC neurons.

### Regulation of ARC POMC neuronal activity by TRPM2 channels

**Fig. 3:**
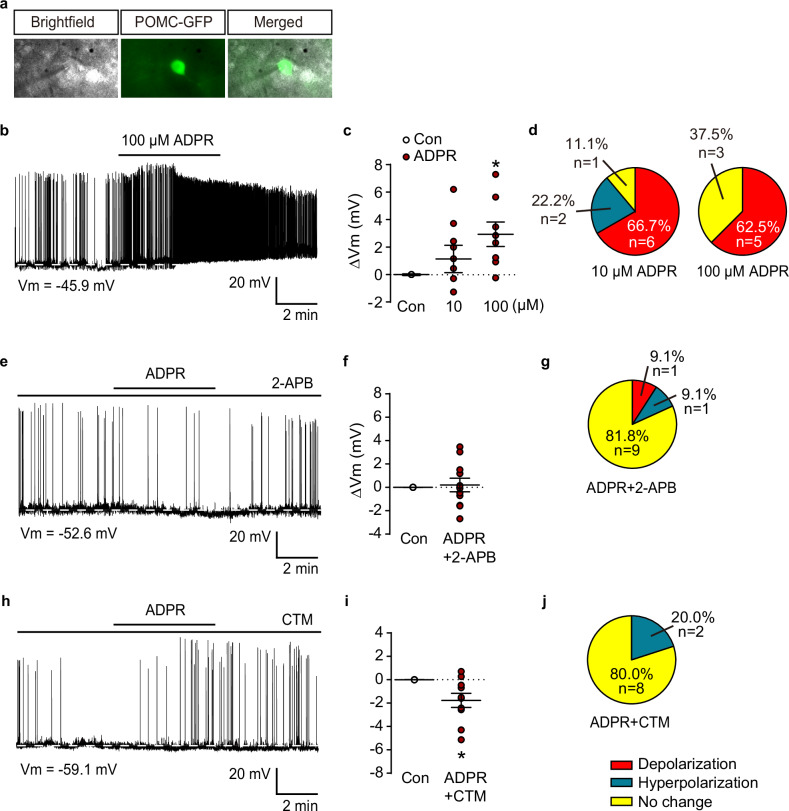
Alterations of neuronal activity of ARC POMC neurons after treatment with the TRPM2 agonist ADPR in NCD-fed normal mice. **a** Representative brightfield, infrared and merged images for establishing a whole-cell patch-clamp recording. **b** Representative recording trace for changes in membrane potential of POMC neurons after ADPR (10, 100 μM, respectively) treatments (aCSF containing 5 mM glucose). Scale bar, 20 mV, 2 min. **c** Pooled data showing POMC neuron depolarization after 10 and 100 μM ADPR treatment. **d** Percentile analyses of response patterns of POMC neurons after 10 μM (left) or 100 μM (right) ADPR treatments. **e** Representative recording trace for changes in membrane potential of POMC neurons after ADPR (100 μM) treatment in the presence of 2-APB. Scale bar, 20 mV, 2 min. **f** Pooled data showing blockade of ADPR-induced depolarization of POMC neurons by 2-APB. **g** Percentile analyses of response patterns of POMC neurons after 100 μM ADPR treatment in the presence of 2-APB. **h** Representative recording trace for changes in membrane potential of POMC neurons after ADPR (100 μM) treatments in the presence of CTM. Scale bar, 20 mV, 2 min. **i** Pooled data showing blockade of ADPR-induced depolarization of POMC neurons by CTM. **j** Percentile analyses of response patterns of POMC neurons after 100 μM ADPR treatment in the presence of CTM. All data are shown as mean ± s.e.m. *P < 0.05 versus control.

As a nonselective cation channel, TRPM2 leads to neuronal depolarization through the influx of Na^+^ and Ca^2+^ ions^32^. To investigate whether TRPM2 induces depolarization in POMC neurons, we used ADPR, the most potent TRPM2 activator^33^. We measured the membrane potential of POMC neurons in the ARC after treatment with either 10 µM or 100 µM ADPR (Fig. 3a). The results revealed that the higher concentration of ADPR (100 µM) induced depolarization in a more significant proportion of POMC neurons (five of eight neurons) than did the lower concentration (10 µM; six of nine neurons). Therefore, we used 100 µM ADPR in subsequent experiments (Fig. 3b–d and Supplementary Table 1). While the membrane potential was significantly depolarized upon treatment with 100 μM ADPR, the membrane potential changes (ΔV) were 2.9 ± 0.9 mV.

**Fig. 4:**
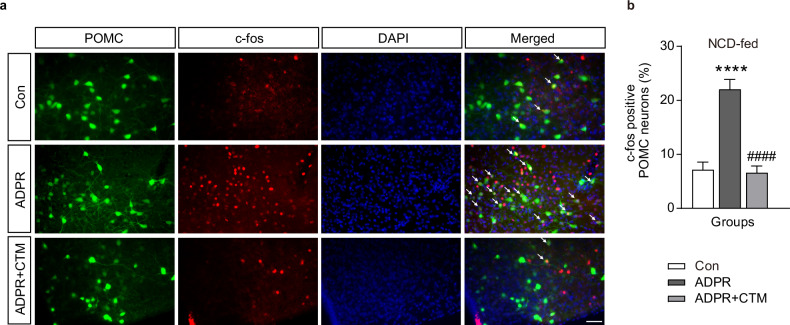
Activation of arcuate (ARC) POMC^TRPM2^ neurons after exposure to ADPR in NCD-fed mice. **a** Fluorescence microscopy images showing c-Fos-positive (red) POMC neurons (green) after an intracerebrovascular injection of ADPR or ADPR with CTM. Scale bar, 50 μm. **b** Pooled data showing the percentage of c-Fos-positive ARC POMC neurons in mice given vehicle, ADPR and ADPR + CTM, respectively. All data are shown as mean ± s.e.m. ****P < 0.0001 versus control; ^####^*P* < 0.0001 versus ADPR.

Next, we confirmed whether TRP channels regulated this effect. The membrane potential of POMC neurons increased significantly after treatment with 100 μM ADPR, and this increase was attenuated by the universal TRP channel blocker 2-aminoethoxydiphenyl borate (2-APB) (Fig. 3e–g and Supplementary Table 1; Δ*V* = 0.2 ± 0.6 mV, *n* = 11 neurons). Similarly, the depolarization of POMC neurons that was increased by ADPR was entirely blocked by CTM pretreatment, and some of the neurons even became hyperpolarized (Fig. 3h–j and Supplementary Table 1). In addition, i.c.v. injection of ADPR (100 μM/μl, 1 μl) increased the expression of c-Fos in POMC neurons; however, when the neurons were pretreated with CTM, the increase in c-Fos caused by ADPR was suppressed (Fig. 4a, b). These results suggest that ADPR-induced TRPM2 activation may regulate POMC neuronal activity.

### ADPR-induced ARC POMC^TRPM2^ activation upregulates BAT and core body temperature in NCD-fed mice

**Fig. 5:**
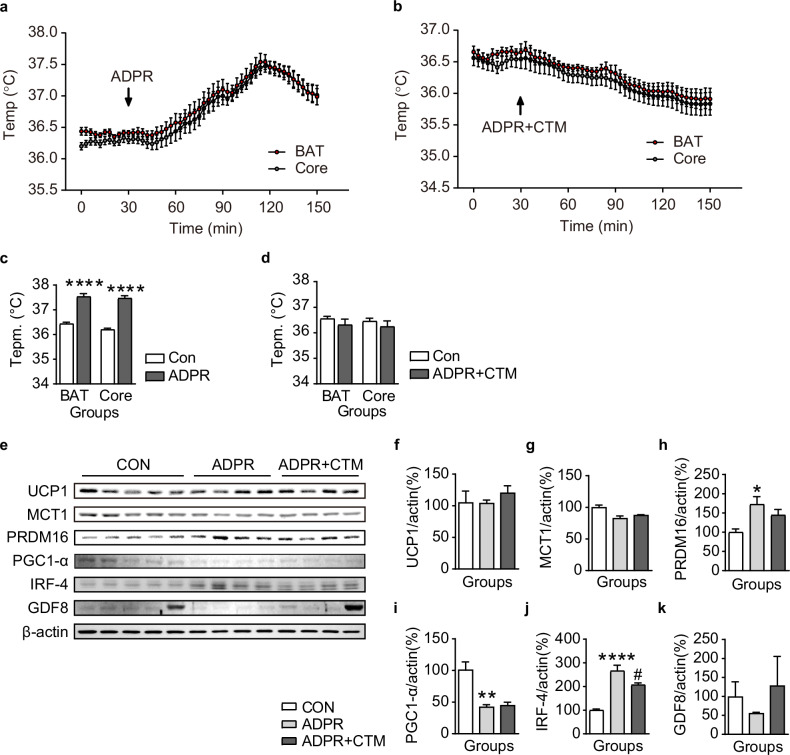
Activation of POMC^TRPM2^ neurons by ADPR increases BAT and core body temperature in NCD-fed mice. **a** Pooled data showing alterations in both BAT and core body temperatures after intracerebroventricular (i.c.v.) injection of ADPR. **b** Pooled data showing alterations in both BAT and core body temperatures after i.c.v. injection of ADPR + CTM. **c**, **d** The plot shows changes in the mean value of both BAT and core body temperature at 0 min (white bar) and 90 min (gray bar) after ADPR treatment (**c**) and ADPR + CTM (**d**). **e** Representative images of western blotting analyses for BAT thermogenic marker proteins after vehicle, ADPR and ADPR + CTM treatments, respectively. **f**–**k** Plots showing changes in expression levels of BAT thermogenic markers, including UCP1 (**f**) MCT1 (**g**) PRDM16 (**h**) PGC1-α (**i**), IRF-4 (**j**) and GDF8 (**k**) after vehicle, ADPR and ADPR + CTZ treatments in NCD-fed mice. All data are shown as mean ± s.e.m. ****P < 0.0001 versus control; ^####^*P* < 0.0001 versus ADPR.

The finding that ADPR treatment increased POMC neuron activity suggested that TRPM2 channels might upregulate BAT and core body temperature, considering that the neural networks of BAT and POMC neurons originate from the ARC. To investigate this hypothesis, we measured BAT and core body temperature in mice after i.c.v. injection of ADPR. As shown in Fig. 5a, b and Supplementary Table 2, i.c.v. injection of ADPR significantly increased both BAT (from 36.4 ± 0.1 °C to 37.5 ± 0.1 °C, *n* = 8) and core body temperature (from 36.2 ± 0.1 °C to 37.5 ± 0.1 °C, *n* = 8). In addition, blockade of TRPM2 by CTM cotreatment with ADPR abolished ADPR upregulation of BAT (from 36.6 ± 0.1 °C to 36.3 ± 0.2 °C, *n* = 8) and core body temperature (from 36.5 ± 0.1 °C to 36.2 ± 0.2 °C, *n* = 8) (Fig. 5c, d).

We then used western blot analyses and quantitative PCR to examine whether POMC^TRPM2^ alters the expression of BAT thermogenic markers (Fig. 5 and Supplementary Figs. 1 and 3). Among thermogenic marker proteins, the expression of PRDM16 and IRF-4 was significantly increased after ADPR treatment, but UCP1 expression did not change (Fig. 5e–k). In particular, ADPR-induced IRF-4 upregulation was blocked by CTM pretreatment.

Moreover, expression levels of *Prdm16* and *Irf4* mRNA levels were increased by ADPR treatment (Supplementary Fig. 1). These data suggest that acute activation of POMC^TRPM2^ is sufficient to generate heat in the BAT under normal conditions, which may be mediated by IRF-4, but not UCP1.

### ADPR-induced ARC POMC^TRPM2^ stimulation upregulates BAT and core body temperature in HFD-fed mice

**Fig. 6:**
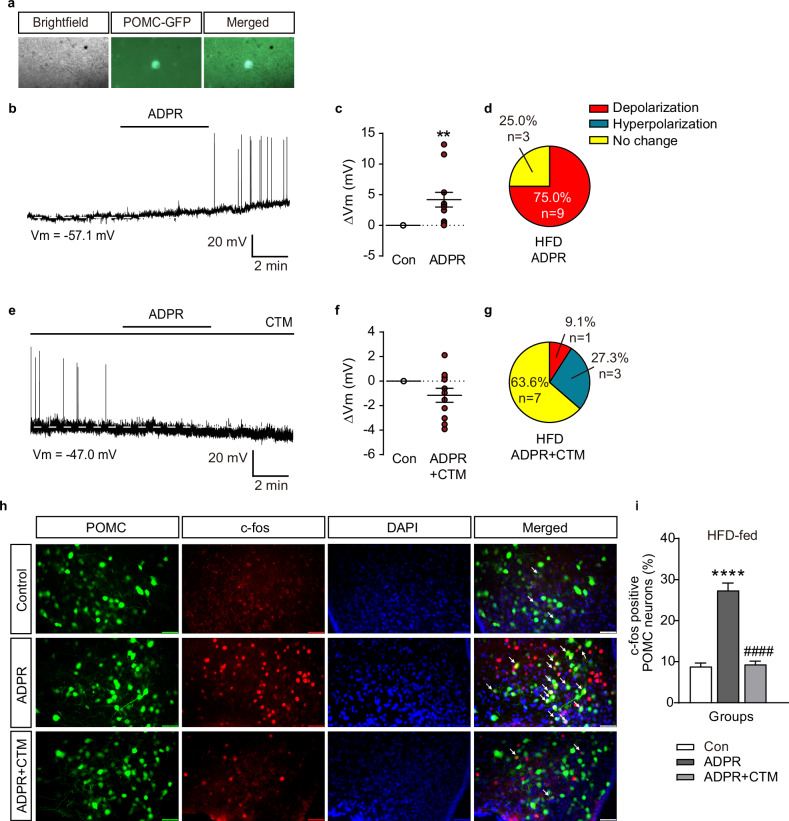
Alterations of neuronal activity of ARC POMC^TRPM2^ after ADPR treatment in HFD-fed mice. **a** Representative brightfield, infrared and merged images for establishing a whole-cell patch-clamp recording. **b** Representative recording trace showing changes in the membrane potential of POMC neurons after 100 μM ADPR treatment in HFD-fed mice. Scale bar, 20 mV, 2 min. **c** Pooled data showing POMC neuron depolarization after ADPR treatment. **d** Percentile analyses of response patterns of POMC neurons after ADPR treatment. **e** Representative recording trace for changes of membrane potential of POMC neurons after ADPR treatments in the presence of CTM. Scale bar, 20 mV, 2 min. **f**, **g** Pooled data (**f**) and percentile analyses of response patterns (**g**) showing blockade of ADPR-induced depolarization of POMC neurons by CTM. **h** Fluorescence microscopy images showing c-Fos-positive (red) ARC POMC neurons (green) in HFD-fed mice after an intracerebrovascular injection of ADPR or ADPR with CTM, respectively. Scale bar, 50 μm. **i** Pooled data showing the percentage of c-Fos-positive ARC POMC neurons in HFD-fed mice given vehicle, ADPR and ADPR + CTM, respectively. All data are shown as mean ± s.e.m. **P < 0.01, ****P < 0.0001 versus control; ^####^*P* < 0.0001 versus ADPR.

To examine whether ARC POMC^TRPM2^ mediated BAT thermogenesis in obesity, we first performed whole-cell patch-clamp recordings using mice fed an HFD for 10 weeks. Similar to the findings in NCD-fed mice, ADPR treatment induced POMC neuron depolarization in the ARC in HFD-fed mice (9 of 12 neurons). The mean value of changes in membrane potential by ADPR treatment was 4.2 ± 1.2 mV. Furthermore, ADPR-induced depolarization of POMC neurons was blocked by CTM pretreatment (Fig. 6a–g and Supplementary Table 1).

**Fig. 7:**
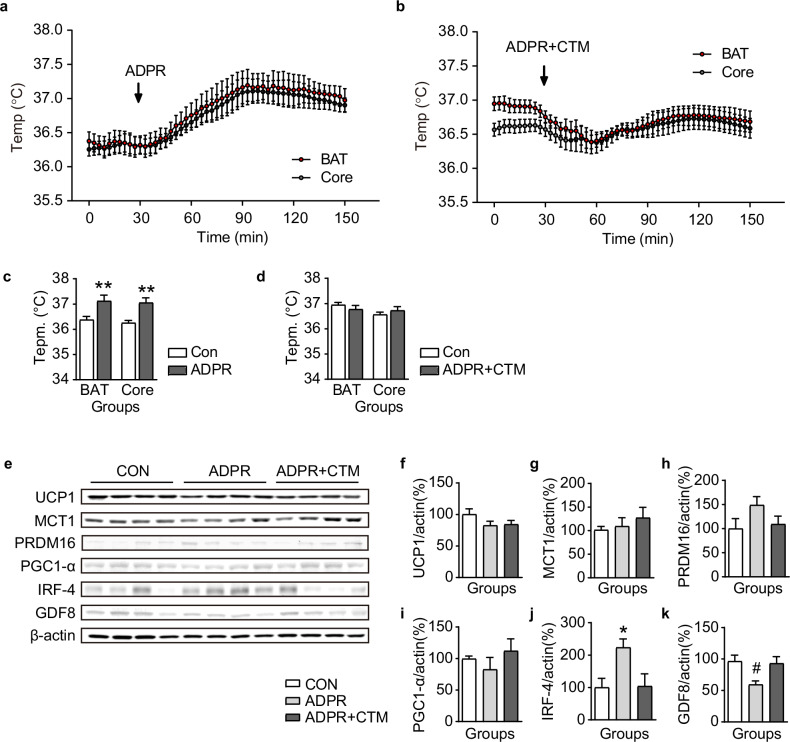
Activation of POMC^TRPM2^ neurons by ADPR increases BAT and core body temperature in HFD-fed mice. **a** Pooled data showing alterations in both BAT and core body temperatures for 150 min after intracerebrovascular injection of ADPR in mice fed an HFD for 12 weeks. **b** Pooled data showing alterations in both BAT and core body temperatures after i.c.v. injection of ADPR + CTM. **c**, **d** Plots showing changes in the mean value of both BAT and core body temperature chosen at 0 min (white bar) for the control period and 90 min (gray bar) after ADPR treatment (**c**) and ADPR + CTM (**d**). **e** Representative images of western blotting analyses for BAT thermogenic marker proteins after vehicle, ADPR and ADPR + CTM treatments. **e**–**k** Plots showing changes in expression levels of BAT thermogenic markers, including UCP1, MCT1, PRDM16, PGC1-α, IRF-4 and GDF8 after vehicle, ADPR and ADPR + CTZ treatments in NCD-fed mice. All data are shown as mean ± s.e.m. *P < 0.05 versus control; ^#^*P* < 0.05 versus ADPR.

To verify these results, IHC was performed using an antibody to c-Fos, a marker of neuronal activity. The expression of c-Fos in POMC neurons of the ARC was significantly increased by i.c.v. injection of ADPR, but was blocked by pretreatment with CTM (Fig. 6h, i). Furthermore, i.c.v. injection of ADPR significantly increased both BAT (from 36.4 ± 0.1 °C to 37.1 ± 0.2 °C, *n* = 6) and core body temperature (from 36.3 ± 0.1 °C to 37.1 ± 0.2 °C, *n* = 6) (Fig. 7a, c and Supplementary Table 2). Blockade of TRPM2 by CTM pretreatment abolished the ADPR-induced increase in BAT (from 36.9 ± 0.1 °C to 36.8 ± 0.1 °C, *n* = 6) and core body temperature (from 36.6 ± 0.1 °C to 36.7 ± 0.2 °C, *n* = 6) (Fig. 7b, d and Supplementary Table 2).

Western blot and quantitative PCR analyses were performed to examine whether POMC^TRPM2^ alters the expression of BAT thermogenic markers in HFD-fed mice (Fig. 7 and Supplementary Figs. 2 and 4). ADPR treatment significantly increased IRF-4 expression (Fig. 7j), and this effect was abolished by CTM pretreatment. In addition, ADPR treatment significantly reduced GDF8 (myostatin) expression, in contrast to its effect on IRF-4 (Fig. 7k).

## Discussion

In this study, we aimed to ascertain whether POMC^TRPM2^ stimulation facilitates energy expenditure via BAT thermogenesis in a murine model. We elucidated the function of POMC^TRPM2^ in terms of changes in the expression of thermogenic markers as well as BAT and core body temperature in both NCD- and HFD-fed mice. Our findings offer new insights into the physiological mechanism of IRF-4-mediated BAT thermogenesis, which is regulated by acute activation of hypothalamic POMC^TRPM2^ neurons.

The extensive discussion in several previous reviews regarding the connection between the melanocortinergic system and BAT thermogenesis highlights well-documented evidence of the mechanism underlying BAT thermogenesis and the involvement of ARC POMC neuronal activation via the sympathetic nervous system^6,34–36^. These results are supported by previous findings that transgenic mice with enhanced leptin and insulin signaling in ARC POMC neurons exhibited a synergistic improvement in glucose metabolism and enhanced energy expenditure, encompassing both BAT thermogenesis and browning of white adipose tissue^37^. Another line of evidence that BAT thermogenesis is increased by stimulation via POMC neurons is that a deficiency in peroxisome proliferator-activated receptor-gamma coactivator-1β in POMC neurons sensitizes leptin-induced BAT thermogenesis. This deficiency also results in elevated body temperature during the satiated state induced by nutrients^38^. In addition, our data suggest that ARC POMC neurons express TRPM2 channels and innervate the BAT (Fig. 1e, f), further supporting the close association between POMC neuronal activity and BAT thermogenesis. Furthermore, we provide direct evidence that ARC POMC neuron-specific stimulation using Gq-DREADD is sufficient to modulate BAT and core body temperature, and the TRPM2 channel is critical for POMC neuronal activation and POMC-mediated BAT thermogenesis (Fig. 2 and Supplementary Fig. 6). Therefore, POMC^TRPM2^ neurons in the ARC are essential in promoting energy expenditure by regulating BAT and core body temperature.

Several studies have proposed that TRP channels regulate energy balance by being expressed on ARC POMC neurons. For instance, TRPC channels mediate leptin-induced POMC excitation, but this effect is abolished by both general TRP channel blockers, La^3+^ and 2-APB^39^. TRPV1, commonly known as the vanilloid receptor, is expressed in POMC neurons and regulates energy balance by modulating their neuronal activity^40^.

However, the thermoregulatory role of TRPM2 in the brain remains controversial. The subset of TRPM2-expressing neurons in the POA of the hypothalamus plays an opposing role, sensing hyperthermia and downregulating body temperature to maintain temperature homeostasis^17–19^. Nevertheless, the ARC, a circumventricular organ involved in sensing chemicals and nutrients, is located near the third ventricle^41^. As an anatomical characteristic, the ARC has a less strict blood–brain barrier. In addition, POMC neurons in the ARC could detect blood glucose levels and temperature changes as primary-order neurons because they expressed thermosensory channels^40^. As shown in Fig. 1, around 80% of ARC POMC neurons express *Trpm2* mRNA, and ARC POMC neurons are colocalized with TRPM2 channels (Fig. 1b, c). In addition, ARC POMC neurons are activated by ADPR treatment, resulting in an increase in BAT and core body temperature, a response that was inhibited by the TRPM2 antagonist CTM (Fig. 5a–d). These data strongly suggest that TRPM2 in the ARC responds to i.c.v.-injected ADPR earlier than does the POA of the hypothalamus. In fact, the degree of increase in BAT and core body temperature induced by ADPR is far from hyperthermia. As shown in Supplementary Table 2, the mild increase in body temperature is within the physiological range. Therefore, our results suggest that activating TRPM2 channels is essential for regulating POMC neuronal activity and subsequent POMC-mediated BAT thermogenesis.

The sympathetic nervous system has been reported to mediate BAT thermogenesis regulation via the ARC neurons of the hypothalamus. For instance, stimulation of ARC POMC neurons with *N*-methyl D-aspartate increased sympathetic nerve activity and the temperature of the BAT via the PVN and dorsomedial nucleus^42^. The PVN is closely connected to the ARC and forms a melanocortinergic pathway, and when activated by α-MSH released from ARC POMC neurons, it plays a role in suppressing food intake and promoting energy expenditure via activation of sympathetic nerve activity^43–45^. Based on our data, approximately 80% of POMC neurons express TRPM2 (Fig. 1), and ADPR, an agonist of TRPM2, depolarized the POMC neurons (Figs. 3 and 4). In particular, activation of POMC neurons by Cre-dependent ARC POMC neuron-specific expression of Gq-coupled DREADDs was sufficient to induce BAT thermogenesis via sympathetic neuronal connections with the BAT (Fig. 2f, g). Also, the increase in BAT temperature caused by chemogenetic stimulation of POMC neurons was suppressed by pretreatment of TRPM2 inhibitor (Fig. 2h, i). Furthermore, when a β3-adrenergic receptor antagonist was administered subcutaneously to inhibit BAT activity via the sympathetic nervous system, the elevation in BAT temperature induced by ADPR was blocked (Supplementary Fig. 5). These data indicate that TRPM2 regulates POMC neurons, which innervate BAT through a neural circuit, possibly via the sympathetic nervous system. Given this, our findings also indicate that TRPM2 knockdown in the ARC is associated with reduced BAT thermogenesis and diminished POMC neuronal activity (Supplementary Fig. 6). However, because the siRNA-mediated knockdown lacked cell-type specificity, we cannot definitively attribute these effects to TRPM2 in POMC neurons alone. Thus, while TRPM2 appears to contribute to thermoregulatory processes within the ARC, further studies using POMC neuron-specific TRPM2-knockout models are necessary to establish its cell-autonomous role in POMC neurons.

Among several significant factors for energy expenditure, adaptive (nonshivering) thermogenesis, also known as BAT-mediated thermogenesis, occurs in response to reduced ambient temperature and overfeeding, making BAT a central thermal and energy homeostasis regulator^46,47^. As a BAT thermogenic marker, UCP1 is exclusively expressed in BAT and is considered essential for BAT-driven nonshivering thermogenesis^46,47^. Indeed, BAT activation by either cold exposure or β-adrenergic agonists, which indicates sympathetic nerve activation, is associated with weight loss and improved glucose and lipid homeostasis caused by UCP1 upregulation, consequently enhancing substrate consumption and thermogenesis^48^. However, recent studies have demonstrated that BAT thermogenesis operates independently of UCP1. For instance, norepinephrine administration significantly increased BAT temperature even in *Ucp1*^−/−^ mice^49^. Moreover, thermogenic factors regulated by cold exposure or cAMP were discovered before UCP1 induction^50,51^. Thus, β-adrenergic signaling continues despite ablation of UCP1. In other words, it is plausible that BAT could generate heat without UCP1.

ADPR administration increased the expression of thermogenic markers, including IRF-4 and PRDM16, without altering the expression of UCP1 and PGC-1α in mice fed NCD (Fig. 5h,j). Similar results were observed regarding the alteration of IRF-4 expression in the mice fed HFD, but the expression level of PRDM16 was not altered (Fig. 7h, j). Interestingly, myostatin expression levels were significantly decreased in the HFD-fed group. IRF-4 is known to affect not only BAT thermogenesis, but also muscle function. In addition, PRDM16 is essential in the differentiation of brown adipocytes from myoblasts and represses muscle differentiation^50^. Recently, Kong et al. demonstrated that IRF-4 induces BAT thermogenesis, but the loss of IRF-4 reduced this thermogenesis by secreting myostatin and decreasing muscle function^51^. These results showed that acute activation of POMC neurons by TRPM2 causes BAT thermogenesis in an IRF-4-dependent manner. In addition, because BAT shares its origin with skeletal muscle, PRDM16 here seems to play a role not only in BAT thermogenesis but also in preventing the loss of BAT from the muscle function promoted by IRF-4. However, BAT thermogenesis induced by acute activation of POMC^TRPM2^ appears to be specifically regulated by IRF-4 rather than by PRDM16. Moreover, the roles of IRF-4 and myostatin secreted from BAT in regulating muscle function are opposing, and loss of IRF-4 is known to induce myostatin secretion^48^. Moreover, the decrease in BAT activity due to obesity is known to increase serum myostatin and reduce muscle production^52^. However, it is assumed that, in obesity, the increase in BAT activity by POMC^TRPM2^ would increase the expression of IRF-4 and, thus, reduce the expression of myostatin, as shown in Fig. 7k. In addition, IRF-4, along with other thermogenic factors, appears to be intricately involved in the UCP1-independent BAT thermogenesis mechanism mediated by acute activation of hypothalamic POMC^TRPM2^ (Supplementary Figs. 1 and 2).

In summary, we suggest here that acute activation of POMC^TRPM2^ neurons could modulate BAT thermogenesis. Our retrograde neuronal tracing data, demonstrating innervation from hypothalamic POMC neurons to BAT, support these results, as do recent studies on the sympathetic connections between these structures. Indeed, our results demonstrate that this acute stimulation of hypothalamic POMC^TRPM2^-mediated BAT thermogenesis may be associated with several thermogenic factors, notably PRDM16 and IRF-4, but not UCP1. Taken together, our findings provide new insights into the physiological mechanism of BAT thermogenesis that is regulated by hypothalamic POMC^TRPM2^ neurons. These observations may form a basis for developing new therapeutic strategies and preventive measures for combating obesity and metabolic disorders, such as type 2 diabetes, through the enhancement of energy expenditure.

## Supplementary information

Supplementary Information
