# Supplementary material for: The potential role of hypothalamic POMCTRPM2 in interscapular BAT thermogenesis
Source: Exp Mol Med. 2025 Sep 12;57(9):2040–51. doi: 10.1038/s12276-025-01538-6 (PMC12508090; doi:10.1038/s12276-025-01538-6)
Supplement: Supplementary file 1 — Supplementary Information [file 12276_2025_1538_MOESM1_ESM.pdf]

The Potential Role of Hypothalamic POMC^{TRPM2} in Interscapular BAT Thermogenesis

Ju Hwan Yang[1], Arbi Bahtiar Boedi Iman Halanobis[1,5], Eun-Hye Byeon[1], Na Hyun Park[1], Sang Won Park[2,5], Hyun Joon Kim[3,5], Dawon Kang[1,5], Deok-Ryong Kim[4,5], Jinsung Yang[4,5], Eun Sang Choe[6], Wanil Kim[4,5*] and Dong Kun Lee[1,5*]

[1] Department of Physiology, Institute of Medical Sciences, Gyeongsang National University College of Medicine, Jinju, 52727, Republic of Korea

[2] Department of Pharmacology, Institute of Medical Sciences, Gyeongsang National University College of Medicine, Jinju, 52727, Republic of Korea

[3] Department of Anatomy, Institute of Medical Sciences, Gyeongsang National University College of Medicine, Jinju, 52727, Republic of Korea

[4] Department of Biochemistry, Institute of Medical Sciences, Gyeongsang National University College of Medicine, Jinju, 52727, Republic of Korea

[5] Convergence of Medical Sciences, Gyeongsang National University College of Medicine, Jinju, 52727, Republic of Korea

[6] Department of Biological Sciences, Pusan National University, Busan, 46241, Republic of Korea

*Corresponding authors: Wanil Kim (wkim@gnu.ac.kr) and Dong Kun Lee (dklee@gnu.ac.kr)

Materials and Methods

Real-time PCR

For real-time PCR analysis, total RNA was extracted from frozen iBAT using TRIzol reagent, following the manufacturer's instructions (Invitrogen). Subsequently, the extracted total RNA from BAT was subjected to RT reaction using RevertAid First Strand cDNA Synthesis Kit (Thermo Scientific). The qPCR reactions were prepared in a final volume of 20 μl, containing 1 μl of 1/10 diluted cDNA, 6 μl of 2X Rotor-Gene SYBR Green master mix (Qiagen) in the presence of primers at 0.5 μM. Real-time PCR analysis was performed using a QiAquant 96 (Qiagen). The 18S ribosomal RNA was used as an internal positive control for normalization of each sample. The primer information used for qPCR is provided in Supplementary Table 3.

Preparation and microinjection procedures for TRPM2 siRNA

TRPM2 siRNA was purchased from Santa Cruz Biotechnology. For delivery, the TRPM2 siRNA was mixed with Invivofectamine (Invitrogen) according to the manufacturer's instructions. Mice were anesthetized with Avertin, and sterile custom guide cannulas (RWD) were stereotaxically implanted into the arcuate nucleus (ARC; AP, −1.5 mm; ML, ±0 mm; DV, −5.5 mm) under aseptic conditions. The mice were allowed to recover for at least three days following surgery. TRPM2 siRNA (1 μg/μl) was administered into the ARC via the guide cannula once daily for five consecutive days. On the sixth day, interscapular BAT and core body temperatures were measured. After temperature assessment, brain tissues were collected for Western blot analysis.

Supplementary Figures

Supplementary Figure 1

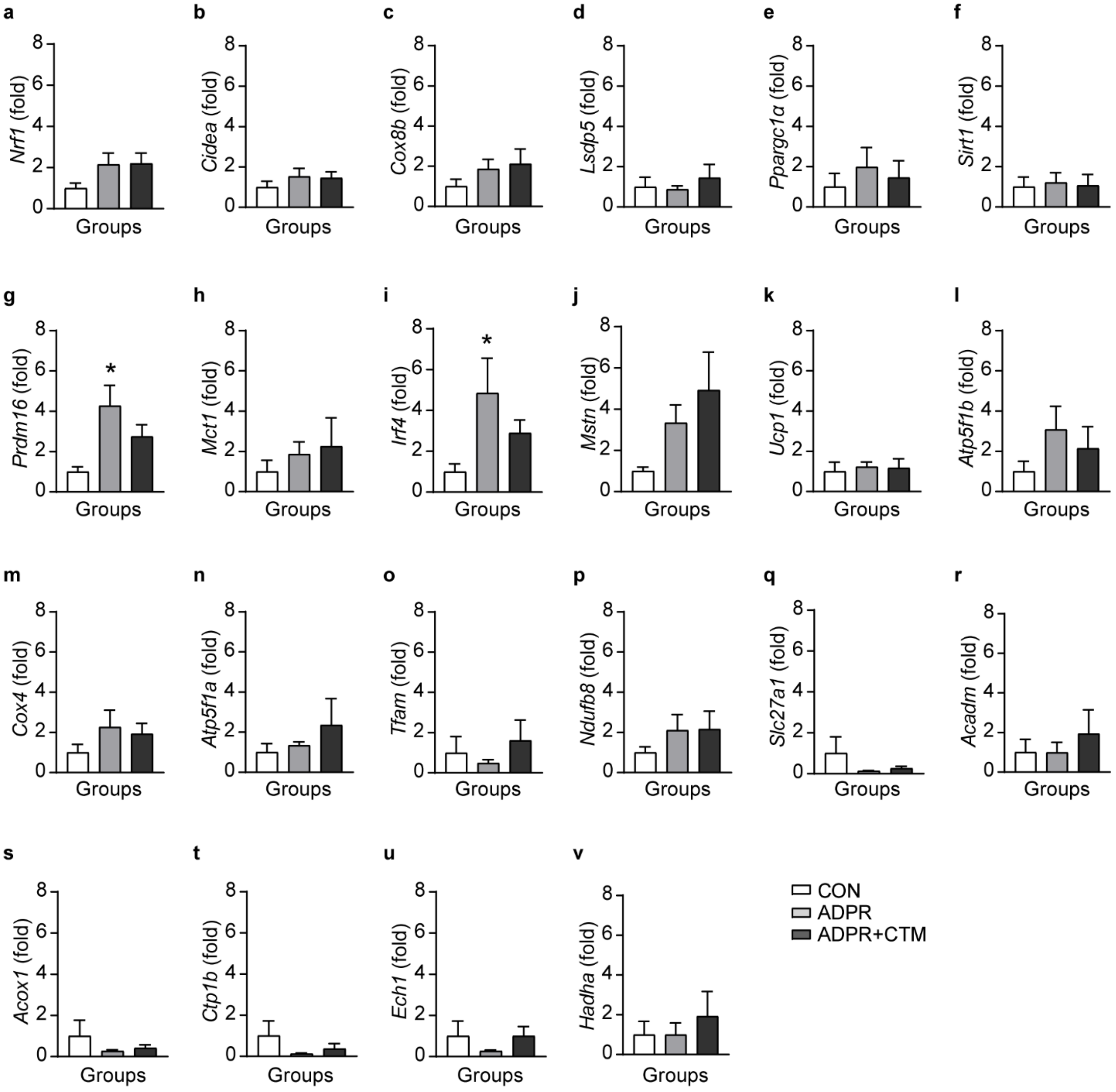

Supplementary Fig 1. Altered expression of thermoregulatory genes in the brown adipose tissue (BAT) after activation of proopiomelanocortin (POMC)^{TRPM2} neurons by adenosine diphosphoribose (ADPR) in normal chow-diet (NCD)-fed mice. (a–v) Summary plots showing quantitative analyses of mRNA of thermoregulatory genes in the BAT after activation of hypothalamic POMC^{TRPM2} neurons by intracerebrovascular injection of ADPR in NCD-fed mice. All data are shown as mean ± standard error of the mean. *$p < 0.05$ vs. control.

Supplementary Figure 2

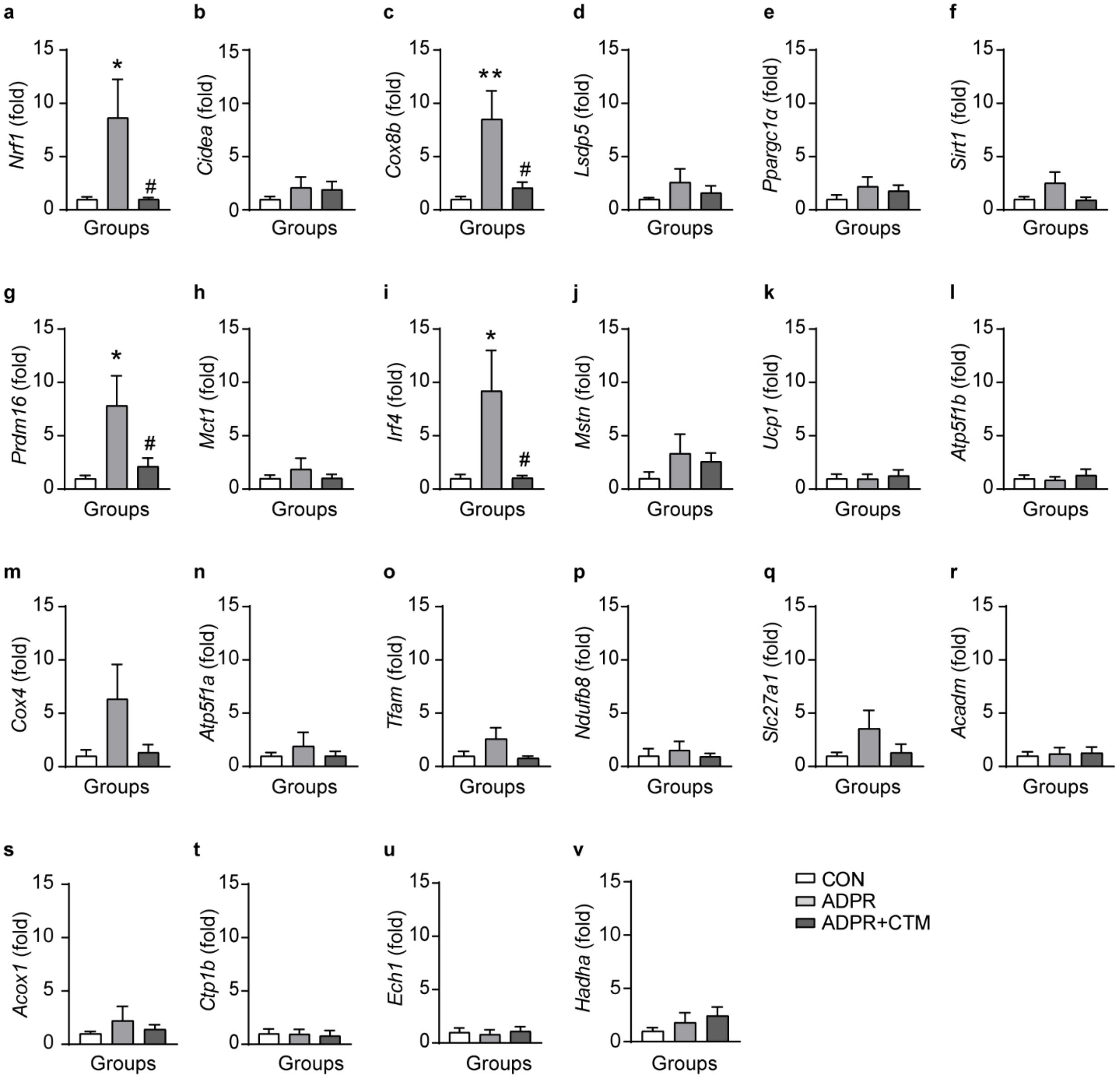

Supplementary Fig 2. Altered expression of thermoregulatory genes in the brown adipose tissue (BAT) after activation of proopiomelanocortin (POMC)^{TRPM2} neurons by adenosine diphosphoribose (ADPR) in high-fat diet (HFD)-fed mice. (a–v) Summary plots showing quantitative analyses of mRNA of thermoregulatory genes in the BAT after activation of hypothalamic POMC^{TRPM2} neurons by intracerebrovascular injection of ADPR in HFD-fed mice. All data are shown as mean ± standard error of the mean. *p < 0.05 vs. control. **p < 0.01 vs. control; #p < 0.05 vs. ADPR.

Supplementary Figure 3

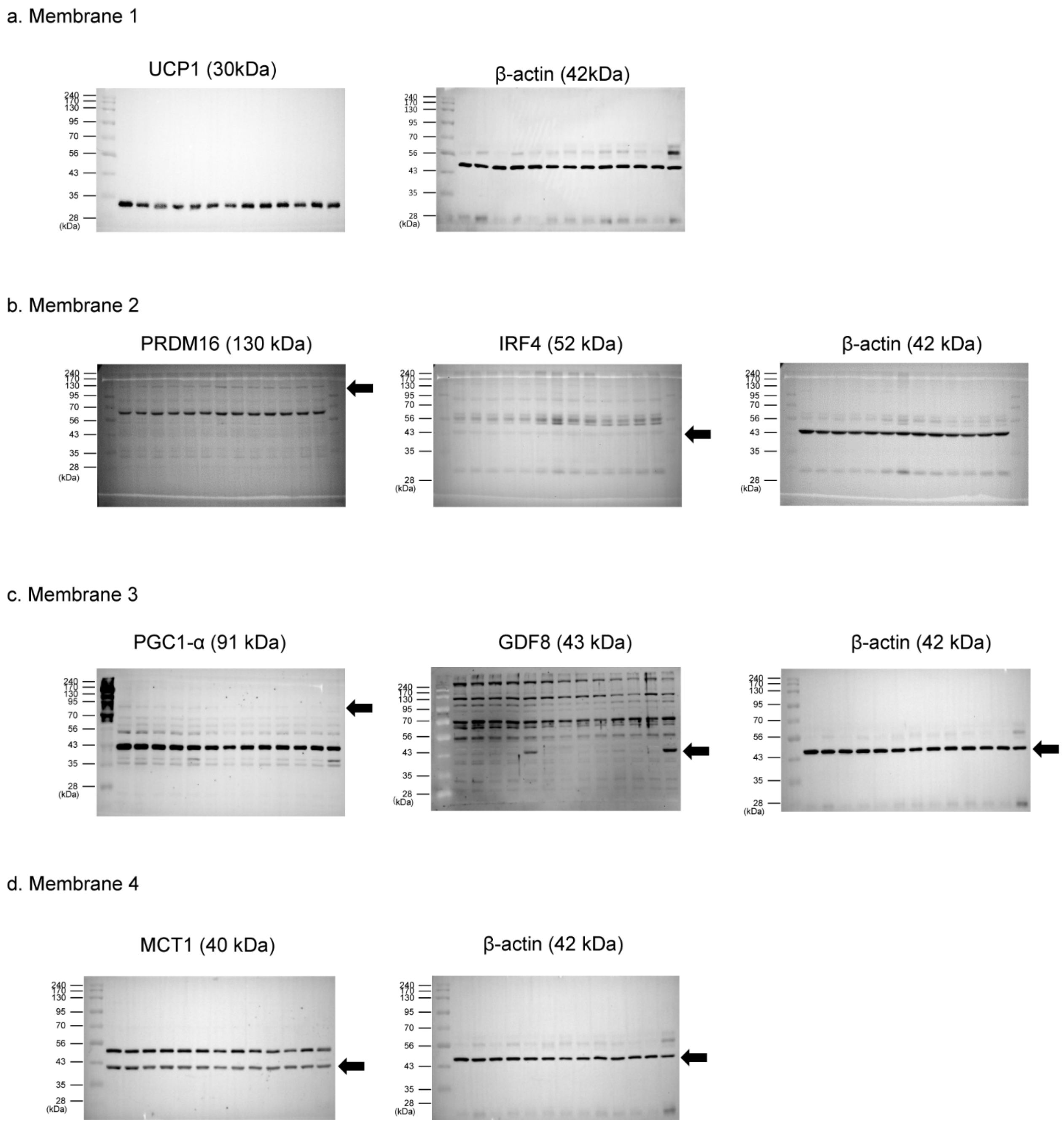

Supplementary Fig. 3. The full-length and whole Western blot images correspond to Fig 5.

Supplementary Figure 4

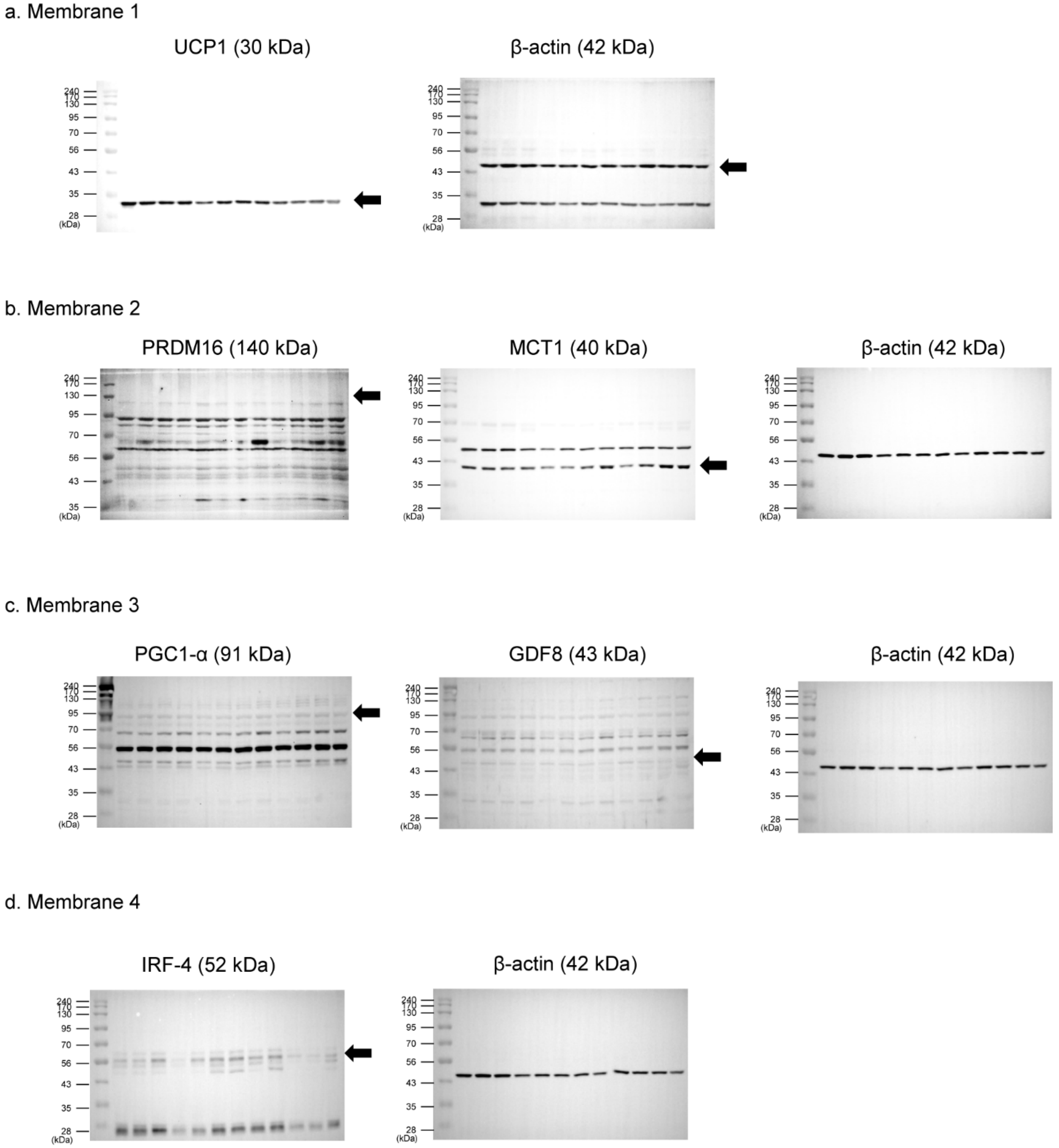

Supplementary Fig. 4. The full-length and whole Western blot images correspond to Fig 7.

Supplementary Figure 5

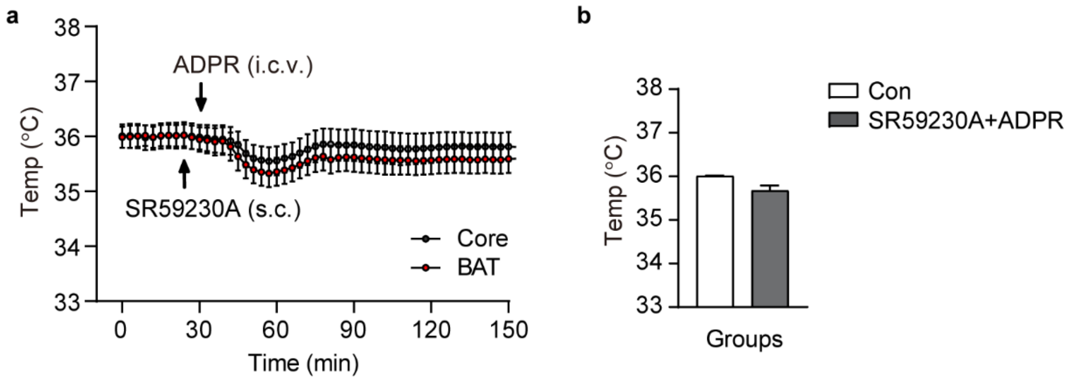

Supplementary Fig 5. Blockade of ADPR-induced BAT and core body thermogenesis after subcutaneous injection of β3-adrenergic receptor antagonist SR59230A. (a) Pooled data showing alterations in both ADPR-induced BAT and core body temperatures for 150 min after blockade of β3-adrenergic receptors in mice fed an NCD for 12 weeks. **(b)** Pooled data showing alterations in both BAT and core body temperatures after blockade of β3-adrenergic receptors. i.c.v. injection of ADPR did not alter the BAT and core body temperatures after the blockade of β3-adrenergic receptors. These results indicate that activation of the sympathetic nervous system through POMC neurons is involved in thermogenesis in BAT.

Supplementary Figure 6

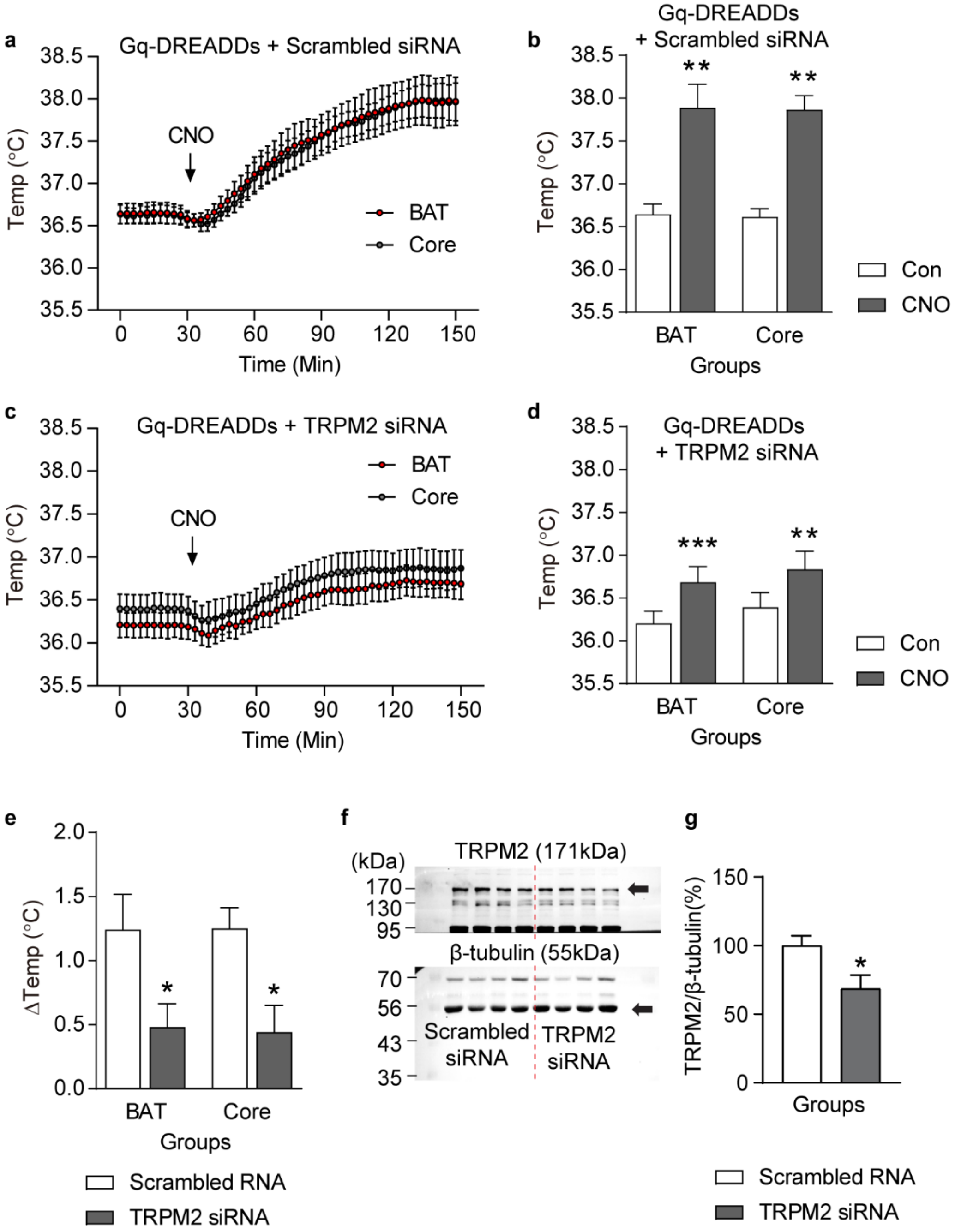

Supplementary Fig 6. Local injection of TRPM2 siRNA into the ARC decreases BAT thermogenesis mediated by POMC neurons. (a) Pooled data showing alterations in both BAT and core body temperatures following i.p. injection of CNO (10 μM) in Gq-DREADD infected mice locally injected with scrambled siRNA into the ARC (n = 4). **(b)** The plot shows the mean changes in both BAT and core body temperature at 0 min (white bar) and 90 min (gray bar)

following CNO treatment, respectively. **(c)** Pooled data showing alterations in both BAT and core body temperatures following i.p. injection of CNO in Gq-DREADD infected mice locally injected with TRPM2 siRNA into the ARC (n = 5). **(d)** The plot shows the mean changes in both BAT and core body temperature at 0 min (white bar) and 90 min (gray bar) following CNO treatment, respectively. **(e)** Comparison of BAT and core body temperature changes induced by POMC neuron-specific activation via CNO in Gq-DREADD-infected mice, following local administration of scrambled siRNA or TRPM2 siRNA, respectively. (**f**) The full-length and whole Western blot gel images of changes in TRPM2 protein expression in the hypothalamus after local administration of both scrambled RNA and TRPM2 siRNA into the ARC. **(g)** Plots showing changes in TRPM2 expression levels following local injection of scrambled RNA or TRPM2 siRNA into the ARC. **p* < 0.05, ***p* < 0.01, ****p* < 0.001 vs. control or scrambled siRNA; ####p < 0.0001 vs. ADPR.

Supplementary Tables

Supplementary Table 1. Summary of changes in membrane potential (ΔmV) before and after drug treatment.

	Drugs		Before (mV)	After (mV)	ΔmV (After-Before)	p value	N	Depolarization (%, n)	Hyper -polarization (%, n)	no change (%, n)
NCD	CNO		-55.5 ± 3.4	-50.9 ± 3.5	4.6 ± 0.9	0.0007***	9	100.0%, n=9	-	-
	ADPR	10 μM	-47.7 ± 2.7	-46.6 ± 2.4	1.1 ± 1.0	0.5377	9	66.7%, n=6	22.2%, n=2	11.1%, n=1
		100 μM	-49.3 ± 2.1	-46.4 ± 2.0	2.9 ± 0.9	0.0330*	8	62.5%, n=5	0%, n=0	37.5%, n=3
	ADPR	2-APB	-51.0 ± 2.2	-50.8 ± 2.3	0.2 ± 0.6	0.7344	11	9.1%, n=1	9.1%, n=1	81.8%, n=9
		CTM	-52.7 ± 2.5	-54.4 ± 2.5	-1.7 ± 0.6	0.016*	10	0%, n=0	20.0%, n=2	80.0%, n=8
HFD	ADPR		-51.1 ± 2.1	-46.9 ± 2.1	4.2 ± 1.2	0.0049**	12	75.0%, n=9	0%, n=0	25.0%, n=3
	ADPR+CTM		-47.4 ± 1.4	-48.6 ± 1.2	-1.2 ± 0.6	0.0689	11	9.1%, n=1	27.3%, n=3	63.6%, n=7

Supplementary Table 2. Summary of changes in BAT and core body temperature (℃) before and after drug treatment.

	Drugs	BAT temp. (℃)		p value	Core body temp. (℃) (%, n)		p value	N
		Before	After		Before	After		
NCD	CNO	36.4 ± 0.1	37.5 ± 0.1	<0.0001****	36.2 ± 0.1	37.5 ± 0.1	<0.0001****	8
	CNO+CTM	36.4 ± 0.1	36.1 ± 0.2	0.0096**	36.5 ± 0.1	36.2 ± 0.2	0.0288*	9
	CNO+scrambled siRNA	36.7 ± 0.1	37.9 ± 0.3	0.0098**	36.6 ± 0.2	37.9 ± 0.2	0.0029**	4
	CNO+TRPM2 siRNA	36.2 ± 0.1	36.7 ± 0.2	0.0009***	36.4 ± 0.2	36.8 ± 0.2	0.0035**	5
	ADPR	36.4 ± 0.1	37.5 ± 0.1	<0.0001****	36.2 ± 0.1	37.5 ± 0.1	<0.0001****	8
	ADPR+CTM	36.6 ± 0.1	36.3 ± 0.2	0.4273	36.5 ± 0.1	36.2 ± 0.2	0.4434	8
HFD	ADPR	36.4 ± 0.1	37.1 ± 0.2	0.002**	36.3 ± 0.1	37.1 ± 0.2	0.0016**	6
	ADPR+CTM	36.9 ± 0.1	36.8 ± 0.1	0.2033	36.6 ± 0.1	36.7 ± 0.2	0.4383	6

Supplementary Table 3. Primer sets that are analyzed in this study.

Gene name	Sequence (5'→3')		Length (bp)	Refseq #
Trmp2	Forward	GCAGCTCCTTGTCCACCTTAT	274	NM_001411900.1
	Reverse	AATGTTGGAGGCTTCACCCC		
18s rRNA	Forward	CTCAACACGGGAAACCTCAC	110	NR_003278.3
	Reverse	CGCTCCACCAACTAAGAACG		
Irf4	Forward	TGCTGGCTTTAGCCTGGTAT	124	NM_001347508.1
	Reverse	GCGTTTCTCTGTGAGTGGAC		
Mstn	Forward	AAGGTGACAGACACACCCAA	124	NM_010834.3
	Reverse	AGTCCCATCCAAAGGCTTCA		
Prdm16	Forward	CGTCCATCGCGGAGAAATAC	184	NM_027504.3
	Reverse	TTAGGCAGGAACTGGAAGGG		
Nrf1	Forward	AGCCACGTTGGATGAGTACA	84	NM_001164226.2
	Reverse	AAGACAGGGTTGGGTTTGGA		
Cidea	Forward	TGGTGGACACAGAGGAGTTC	112	NM_007702.2
	Reverse	TGCTTGCAGACTGGGACATA		
Cox8b	Forward	TTCCCAAAGCCCATGTCTCT	124	NM_007751.3
	Reverse	AAGTGGGCTAAGACCCATCC		
Lsdp5	Forward	ATGCTCTACAGGCCACCTTT	129	NM_001077348.1
	Reverse	GCAGGACCAAATCCAGGAAC		
Ppargc1α	Forward	ACAACGCGGACAGAATTGAG	170	NM_001402987.1
	Reverse	GTTTCGTTCGACCTGCGTAA		
Sirt1	Forward	AGTAAGCGGCTTGAGGGTAA	117	NM_001159589.2
	Reverse	GCCACAGGAACTAGAGGACA		
Mct1	Forward	CTTCATTGGCATGGGCATCA	140	NM_009196.4
	Reverse	ACTGTGCAGCTTTCATCGTC		
Ucp1	Forward	TGTTGTCTTCAGGGCTGAGT	88	NM_009463.3
	Reverse	CTTCGGAAGTTGTCGGGTTC		
Atp5f1b	Forward	CTGGTTTGACCGTTGCTGAA	118	NM_016774.3
	Reverse	CTGCCCAATAAGGCAGACAC		
Cox4	Forward	CAAGCGAATGCTGGACATGA	91	NM_001293559.1
	Reverse	GGGCTCTCACTTCTTCCACT		
Atp5f1a	Forward	GAGCTGACACGTCTGTTGAC	118	NM_007505.2
	Reverse	CTCCAACATTGTCGGGTTCC		
Tfam	Forward	GTTGTTGGATGGCATGGGTT	117	NM_009360.4
	Reverse	CGTGTAGAGCTCACGTCTCT		
Ndufb8	Forward	GGTGCTGTCTCAAGAAGCTG	163	NM_001347447.1
	Reverse	AATGTCTGAGCACCACATGC		

Slc27a1	Forward	CTACCACTCTGCAGGGAACA	160	NM_001357180.1
	Reverse	CAGGTAGCGGCAGATTTCAC		
Acadm	Forward	GAAAGCTGCTAGTGGAGCAC	86	NM_007382.5
	Reverse	CTGGTAACTGAGCCTAGCGA		
Acox1	Forward	ATGCCTTCCACTTTCTCGGA	173	NM_001271898.2
	Reverse	GCAAGCCATCCGACATTCTT		
Cpt1b	Forward	CCGACAGAAGCAAACCTGAG	127	NM_009948.2
	Reverse	CTCAGAGCCTCCCGACTAAG		
Ech1	Forward	CACAAGAGGCCTCCAAGAGA	159	NM_016772.1
	Reverse	ATTCCACCAACTCCCTCCAG		
Hadha	Forward	CCAGCCCGGAACGAATTAAA	130	NM_178878.3
	Reverse	ACAGACCGTGGAGATTTGGT		
